# Clinical Prognostic Factors Associated with Good Outcomes in Pediatric Bell’s Palsy

**DOI:** 10.3390/jcm10194368

**Published:** 2021-09-24

**Authors:** Myung Chul Yoo, Dong Choon Park, Jae Yong Byun, Seung Geun Yeo

**Affiliations:** 1 Department of Physical Medicine & Rehabilitation, School of Medicine, Kyung Hee University, Seoul 02447, Korea; famousir@naver.com; 2 St. Vincent’s Hospital, The Catholic University of Korea, Suwon 16247, Korea; park.dongchoon@gmail.com; 3 Department of Otorhinolaryngology, Head and Neck Surgery, School of Medicine, Kyung Hee University, Seoul 02447, Korea; otorhino512@naver.com

**Keywords:** prognostic factor, pediatric Bell’s palsy, complete recovery

## Abstract

The prognosis of children with Bell’s palsy remains unclear due to its relatively low incidence, and thus, the small number of patients included in individual studies. To evaluate the prognosis of children with Bell’s palsy and identify the predictive value of specific factors that contribute to complete recovery, a retrospective cohort study was conducted of all patients with Bell’s palsy who visited the outpatient clinic of our university hospital between January 2005 and December 2020. We identified the parameters associated with a favorable recovery after 6 months in pediatric patients with Bell’s palsy. Factors recorded for each patient included age, sex, side affected by palsy, time between symptom onset and start of treatment, treatment methods, and the House–Brackmann grade (H–B) grade. The results of the multivariable analysis revealed that the lower degree of initial facial nerve paralysis presented as H–B grade II–IV was a significant favorable prognostic factor (OR: 3.86; 95% CI: 1.27–11.70; *p* < 0.05). Our results showed that the most important factor influencing the complete recovery of Bell’s palsy in children was the lower initial H–B grade at initial presentation.

## 1. Introduction

Acute peripheral facial palsy is uncommon in children, with an annual incidence ranging from 5 to 21 per 100,000 children [1,2]. Idiopathic facial paralysis, known as Bell’s palsy, is a disease characterized by acute, unilateral, idiopathic peripheral facial palsy [3]. Unilateral facial weakness of unknown cause develops rapidly, with Bell’s palsy considered the most common cause of facial paralysis in children compared with other etiologies, including traumatic, congenital, infectious and neoplastic causes [4,5,6]. The etiology and pathophysiology of Bell’s palsy in children are not completely understood. It can be caused by inflammation and edema of the facial nerve fibers, with infiltration of lymphocytes and associated demyelination or axonal degeneration [7,8]. Moreover, infections with many viruses have been reported to cause acute peripheral facial paralysis in children. These findings have suggested that treatment with corticosteroids and/or antiviral agents may be effective in patients with Bell’s palsy. Corticosteroid treatment of adults was shown to improve their chances of recovery, especially if treatment is started within 72 h of symptom onset [9]. In contrast to adults, the clinical prognosis of Bell’s palsy in children is not well documented. To date, there has been a relative lack of research on the prevalence, treatment, and outcomes of Bell’s palsy in children and no standardized treatment guidelines have yet been developed. Therefore, the treatment of children, including the types and dosages of corticosteroids and antiviral agents, is empirical, based on each physician’s experience.

The natural progression of Bell’s palsy in children is thought to be good, with many children tending to show favorable recovery within two months and most by six months, with spontaneous recovery occurring in up to 90% of children under the age of 14 years [10,11]. However, the degree of paralysis at onset can affect the degree of recovery and, in the case of severe paralysis, patients are rarely known to achieve a complete recovery of the nerve function [12,13]. In addition, several studies have reported that treatment with oral steroids is effective, whereas other studies have shown that treatment with oral steroids does not contribute significantly to recovery in children with Bell’s palsy [14,15]. Although many studies have assessed the causes, diagnosis, treatment and prognosis of adults, fewer studies have assessed these factors in pediatric patients, with clinical evidence of good recovery outcomes currently lacking.

Based on this background, it is important for physicians treating Bell’s palsy in children to predict clinical outcomes in each patient. This retrospective study analyzed outcomes in 88 children with Bell’s palsy, including their rate of recovery and the association between various clinical and demographic parameters. The aim of the present study was to evaluate the clinical features and identify the parameters associated with complete recovery in pediatric patients with Bell’s palsy.

## 2. Materials and Methods

### 2.1. Subjects and Study Design

This retrospective cohort study included the medical records of all children aged <19 years with facial nerve palsy admitted to the Department of Otorhinolaryngology, Head and Neck Surgery of our university hospital between January 2005 and December 2020. The medical records of 133 patients admitted to our university hospital for management of acute facial palsy were reviewed. Patients were included if they had acute, unilateral, isolated lower motor neuron type of facial palsy. Patients were excluded if they had iatrogenic facial nerve palsy such as a birth trauma, neoplasms, Ramsay Hunt syndrome, herpes zoster oticus, and unclear medical records. In addition, patients with an unclear timing of symptom onset and those who could not be followed up for at least 6 months were excluded. This study included 88 patients aged <19 years. Baseline characteristics and outcome measures, including findings on otorhinolaryngological examinations, grading of facial function, age, sex, and previous history of facial palsy, were assessed prior to initiation of treatment.

All patients were admitted to hospital for at least 4 days, and they were started on a treatment with an oral steroid or a steroid combined with an antiviral drug within 7 days from the onset of paralysis. Steroid treatment consisted of a course of oral prednisolone for 12 days, at doses of 1 mg/kg per day for 4 days, followed by tapering by daily reduction depending on the symptoms, and meticulous eye care. Antiviral treatments included acyclovir or famciclovir. Because the 88 patients were recruited over 14 years, the antiviral agent was dependent on date of recruitment. Initially recruited patients were treated with acyclovir (1000–2400 mg/day for 5 days), whereas, at present, patients with Bell’s palsy are treated with famciclovir (750 mg/day for 7 days).

The initial severity of Bell’s palsy was assessed using the H–B grading system by three otolaryngologists with more than 20 years of experience in facial palsy. As evaluating facial paralysis is more difficult in young children than in adults, the severity of Bell’s palsy was based on the observation of the patient’s facial movements while at rest, crying or laughing. Six months after treatment, the extent of recovery in all patients was evaluated by H–B grade, with H–B grade I defined as a complete recovery, and H–B grade ≥ II defined as incomplete recovery.

**Figure 1:**
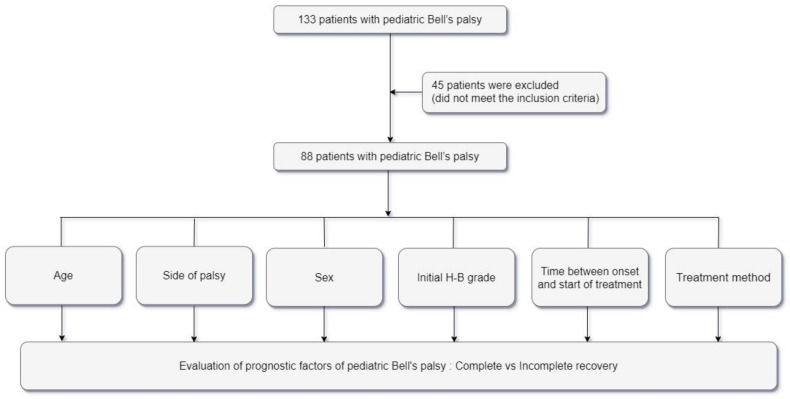
Flow diagram of the study design. H–B grade = House–Brackmann grade.

Factors recorded for each patient included age, side affected by palsy, sex, the degree of initial facial nerve paralysis (initial H–B grade), final H–B grade six months after onset, time between onset and start of treatment, and treatment methods. Initiation of treatment was classified as ≤72 h and >72 h in patients receiving prednisolone with or without antiviral therapy (Figure 1). The protocol of this retrospective study was approved by the Institutional Review Board (IRB No. 2019-07-065), which waived the requirement for written informed consent owing to the retrospective nature of the study.

### 2.2. Statistical Analyses

Categorical variables were reported as number (percentage) and compared by Fisher’s exact test, whereas continuous variables were reported as mean ± standard deviation and compared by the Mann–Whitney U test. Odds ratios (OR) and 95% confidence intervals (CI) were calculated by multivariable Firth’s penalized likelihood logistic regression. All statistical analyses were performed using SAS 9.4 software (SAS Institute Inc., Cary, NC, USA), with *p* < 0.05 defined as statistically significant.

## 3. Results

The baseline demographic and clinical characteristics and outcome measures, according to age group in pediatric patients with Bell’s palsy, are shown in Table 1. Of the 133 children initially included, 45 did not meet the inclusion criteria. The remaining 88 children consisted of 43 (48.9%) boys and 45 (51.1%) girls with Bell’s palsy. Division by age showed that eight patients were aged 0–1 years (mean age, 0.9 ± 0.4 years), 34 were aged 2–10 years (mean age, 6.1 ± 2.6 years) and 46 were aged 11–18 years (mean age, 15.5 ± 2.2 years). Female predominance was observed in patients aged 0–10 years, but male predominance was observed in patients aged 11–18 years. Forty-five patients (51.1%) had right-sided and 43 (48.9%) had left-sided Bell’s palsy. At onset, 77.3% of patients had H–B grade II–IV, whereas 22.7% had H–B grade V–VI. Twenty-seven patients (30.7%) started treatment within 72 h of symptom onset, whereas 61 patients (69.3%) started treatment at a later time. Sixty-five patients (73.9%) were treated with steroid alone, whereas 23 (26.1%) were treated with both a steroid and an antiviral agent. Mean initial H–B grade on admission was 3.61 ± 1.2 and mean final H–B grade at 6 months was 1.57 ± 0.58. Table 2 shows the demographic and clinical characteristics of two groups of patients (complete recovery vs. incomplete recovery). At 6 months, 49 (55.7%) of the 88 patients showed complete recovery, defined as H–B grade I–II. The other 39 patients (44.3%) showed incomplete recovery, defined as H–B grade II–IV. The only factor differing significantly between the complete and incomplete recovery groups was initial lower H–B grade II–IV.

Multivariable logistic regression analysis was performed to identify prognostic factors that affected the final recovery, revealing a single parameter that influenced complete recovery. (Table 3). Complete recovery was associated with a lower initial H–B grade (OR: 3.86; 95% CI: 1.27–11.70; *p* < 0.05). Except for lower initial H–B grade, other variables including sex, side of palsy, age, and onset of treatment did not reveal any significance for predicting the probability of complete recovery. Moreover, the use of oral steroid alone did not show any significant difference in the odds ratio compared with the antiviral therapy in the treatment method, and there was no statistically significant difference.

## 4. Discussion

Prognostic factors in adults with Bell’s palsy have been well defined, with favorable outcomes reported in 80–94% of adult patients treated with or without combination antiviral therapy [16,17,18]. Less is known, however, about clinically prognostic factors in pediatric patients with Bell’s palsy. Thus, there has been a relative lack of research on the prevalence, treatment, and outcomes of Bell’s palsy in children, and no standardized treatment guidelines have yet been developed. The purpose of this study was to identify the parameters associated with complete recovery in pediatric patients with Bell’s palsy. This retrospective cohort study involving 88 pediatric patients with Bell’s palsy who visited an outpatient clinic identified multiple parameters associated with complete recovery after 6 months. Our results showed that the most important factor influencing complete recovery was a lower H–B grade at initial presentation.

Previous studies showed that pediatric patients with Bell’s palsy had a better prognosis than adult patients, suggesting that younger age was associated with a positive prognosis [2,16]. In contrast, other studies in children found that patient age at the time of complete or incomplete paralysis may or may not be associated with patient prognosis. For example, good outcomes were observed in children aged <8 years, but outcomes did not differ significantly between children aged <8 and 8–19 years [19]. Because favorable recovery rates tended to be higher in children than in adults, we hypothesized that, in comparing three age groups (0–1 vs. 2–10 vs. 11–19 years), younger age would be associated with better prognosis. However, we found that recovery rates did not differ significantly in these three groups, indicating that younger age does not significantly affect complete recovery rates in pediatric patients with Bell’s palsy.

Despite the identification of clinical prognostic factors in adults with Bell’s palsy, the relatively low incidence of Bell’s palsy in pediatric patients has limited the ability to identify factors related to patient prognosis. A relatively large number of adult patients with Bell’s palsy has been studied, and many randomized control trials have assessed treatments in these patients [17,20]. In general, the treatment of idiopathic Bell palsy in children has been based on studies in adult populations. Therefore, the treatment of children, including the types and dosages of corticosteroids and antiviral agents, is empirical, based on each physician’s experience. Investigations of large numbers of pediatric patients with Bell’s palsy and relevant evaluations such as randomized control studies are needed, but to date no such studies have been performed [21]. All studies in children have been retrospective case studies, most involving fewer than 100 patients (Table 4). The largest number of pediatric Bell’s palsy patients included in a study was 102 [22]. To our knowledge, the present study is the first to perform multiple logistic regression analysis of various factors that affect complete recovery. We found that lower initial H–B grade (II–IV) was significantly associated with complete recovery. This finding was consistent with studies in adults Bell’s palsy in that a lower degree of initial facial nerve paralysis, as determined by H–B grade, was associated with a favorable outcome. Yoo et al. reported that patients with Bell’s palsy had favorable outcomes at six months when the initial H–B grade was IV or lower [19]. Our findings were consistent with those in adults in that multivariate logistic regression analyses found that the severity of facial palsy, as determined by initial H–B grade II–IV, was the most important factor associated with good prognosis.

The time from symptom onset to treatment and treatment method were not significant prognostic factors in children with Bell’s palsy. American Academy of Otolaryngology-Head and Neck Surgery treatment guidelines recommend that patients aged >16 years with Bell’s palsy be treated within 72 h of symptom onset [9]. Many studies, however, have reported that the time to onset of treatment and the treatment method were not predictive of prognosis in pediatric patients with Bell’s palsy. Although one study recommended that steroid treatment be started within 72 h, preferably within 24 h, another study reported no significant relationship between delayed onset of treatment and recovery rates [22,28]. We found that recovery rates did not differ in patients treated within or after 72 h following symptom onset. Interestingly, the incidence and rate of initiation of treatment within 72 h were low in the present study, suggesting that awareness of the symptoms of Bell’s palsy in children, especially in infants and younger children, may be delayed, resulting in a longer delay in starting treatment than in adults. Despite many of the children in the present study not starting steroid treatment within 72 h, the prognoses were good in all children.

Early steroid therapy has been reported to improve recovery in adults with Bell’s palsy. The American Academy of Otolaryngology-Head and Neck Surgery treatment guidelines suggested that oral steroid treatment within 72 h of symptom onset is likely to be effective in these patients, with or without concurrent antiviral therapy [9]. While there is a consensus that rapid use of steroids is effective in adults, few studies are available in children, and even the recommended dosage for steroid administration is unclear. Arican et al. compared patients treated with two different doses of prednisolone therapy (1 mg/kg/d vs. 2 mg/kg/d), and recovery rates were found to be comparable in patients treated between the two groups [3]. Additional antiviral treatment of Bell’s palsy is based on the hypothesis that a simple herpes virus infection can cause inflammation of the facial nerve. Although treatment with prednisolone alone reduced the time to facial recovery and was sufficient to treat patients with Bell palsy, concurrent antiviral therapy has been reported to enhance recovery when compared with steroids alone [29]. Although it is not clear whether the recovery was due to the good recovery rate in children or resulted from steroid treatment, despite the lack of consistent evidence, the treatment of Bell’s palsy in children was, for the most part, based on studies that were conducted on adult populations. To date, there have been no prospective, randomized, controlled, double-blind studies in children with Bell’s palsy, suggesting a need for these trials.

The rate of complete recovery in the present study was lower than those reported in previous studies (Table 4). Our study was based on data collected over a period of longer than 10 years, with the initial and final severities of Bell’s palsy assessed by three otolaryngologists. Their evaluations of the degree of paralysis of the facial nerve may have resulted in a lower degree of unity than evaluations by a single evaluator. Additionally, numerous studies have indicated that a final H–B grade of II or lower is prognostic of favorable outcomes, as determined by normal function in daily life [30,31]. After excluding 11 patients with initial H–B grade II, we found that 72 (93.5%) of the 77 patients showed favorable recovery, defined as H–B grade I–II at 6 months.

This study has several limitations. First, the significant limitations of the study are its retrospective design and its inclusion of a population with a low incidence of Bell’s palsy and a high recovery rate, resulting in assessment of small numbers of patients. Second, although the initial severity of Bell’s palsy was assessed by three otolaryngologists, they were not blinded, which may have introduced selection bias. Third, we used the H–B grading system to assess the severity of palsy, but we did not evaluate synkinesis, a risk factor for poor outcome and major complications of Bell’s palsy. Fourth, the five patients who did not recover from facial palsy after six months were not followed-up. These limitations may be overcome by a truly randomized study, allowing clinicians to determine treatment methods for patients with idiopathic peripheral facial palsy.

## 5. Conclusions

In conclusion, this retrospective study evaluated factors associated with good outcomes in pediatric patients with Bell’s palsy. Factors such as sex, side of palsy, younger age, onset of treatment and treatment method did not demonstrate statistically significant correlations with good outcomes. A lower degree of initial facial nerve paralysis, as measured using the H–B grading system, was found to be a good prognostic factor affecting the complete recovery of children with Bell’s palsy.

## Figures and Tables

**Table 1:** Baseline demographic and clinical characteristics and outcomes in the three age groups of children with Bell’s palsy.

Variable	Total (*n* = 88)	Age Group of Years		
		Infant 0–1 y (*n* = 8)	Younger Children 2–10 y (*n* = 34)	Older Children 11–18 y (*n* = 46)
Sex, *n* (%)				
Male	43 (48.9)	3 (37.5)	16 (47.1)	24 (52.2)
Female	45 (51.1)	5 (62.5)	18 (52.9)	22 (47.8)
Side of palsy, *n* (%)				
Right	45 (51.1)	5 (62.5)	17 (50.0)	23 (50.0)
Left	43 (48.9)	3 (37.5)	17 (50.0)	23 (50.0)
Age, mean (SD), years	10.5 ± 5.8	0.9 ± 0.4	6.1 ± 2.6	15.5 ± 2.2
Initial H–B grade, *n* (%)				
II–IV	68 (77.3)	6 (75.0)	25 (73.5)	37 (80.4)
V–VI	20 (22.7)	2 (25.0)	9 (26.5)	9 (19.6)
Onset of treatment, *n* (%)				
≤72 h	27 (30.7)	3 (37.5)	7 (20.6)	17 (37.0)
>72 h	61 (69.3)	5 (62.5)	27 (79.4)	29 (63.0)
Treatment method, *n* (%)				
Oral steroids only	65 (73.9)	6 (75.0)	31 (91.2)	28 (60.9)
Combination antiviral therapy	23 (26.1)	2 (25.0)	3 (8.8)	18 (39.1)

H–B grade = House–Brackmann grade; SD = standard deviation. Data are presented as number (%) and mean (SD).

**Table 2:** Baseline demographic and clinical characteristics at admission and outcomes at 6 months according to the severity of Bell’s palsy.

Variable	Total	Complete Recovery Group *	Incomplete Recovery Group **	*p*-Value
	(*n* = 88)	(*n* = 49)	(*n* = 39)	
Sex, *n* (%)				
Male	43 (48.9)	28 (57.1)	15 (38.5)	0.08
Female	45 (51.1)	21 (42.9)	24 (61.5)	
Side of palsy, *n* (%)				
Right	45 (51.1)	28 (57.1)	17 (43.6)	0.21
Left	43 (48.9)	21 (42.9)	22 (56.4)	
Age, years, *n* (%)				
Infant, 0–1	8 (9.1)	4 (8.1)	4 (10.2)	0.99
Younger child, 2–10	34 (38.6)	19 (38.8)	15 (38.5)	
Older child, 11–18	46 (52.3)	26 (53.1)	20 (51.3)	
Initial H–B grade, *n* (%)				
II–IV	68 (77.3)	43 (87.8)	25 (64.1)	0.008
V–VI	20 (22.7)	6 (12.2)	14 (35.9)	
Onset of treatment, *n* (%)				
≤72 h	27 (30.7)	14 (28.6)	13 (33.3)	0.63
>72 h	61 (69.3)	35 (71.4)	26 (66.7)	
Treatment method, *n* (%)				
Oral steroids only	65 (73.9)	36 (73.5)	29 (74.4)	0.93
Combination antiviral therapy	23 (26.1)	13 (26.5)	10 (25.6)	

H–B grade = House–Brackmann grade. Data are presented as number (%) and *p*-value. * Complete recovery was defined as an H–B grade I; ** incomplete recovery was defined as an H–B grade II–IV.

**Table 3:** The results of multiple logistic regression analysis of factors predicting complete recovery in children with Bell’s palsy.

Variable		Probability of Complete Recovery *			
		OR	95% CI *p*-Value		
Sex	Male	2.16	0.87	5.35	0.09
	Female	1.00			
Side of palsy	Right	1.65	0.65	4.18	0.29
	Left	1.00			
Age (years)	Infant (0–1)	1.00			
	Younger child (2–10)	1.13	0.21	6.10	0.89
	Older child (11–18)	0.96	0.19	4.99	0.96
Initial H–B grade	II–IV	3.86	1.27	11.70	0.02
	V–VI	1.00			
Onset of treatment	≤72 h	1.00			
	>72 h	1.28	0.45	3.64	0.64
Treatment method	Oral steroids only	1.00			
	Combination antiviral therapy	1.22	0.40	3.72	0.73

OR = odds ratio; 95% CI = 95% confidence interval; H–B grade = House–Brackmann grade. Data are presented as odds ratio, difference (95% confidence intervals). * Complete recovery was defined as an H–B grade I.

**Table 4:** Studies describing outcomes in pediatric Bell’s palsy.

Authors (Years)	Study Type	Study Population	Mean Age (Years)	Mean Follow-Up Period	Dose of Steroid	Recovery Rate (%)	Outcome Measures
Lee et al. (2020) [23]	Retrospective cohort	53 children with BP	10.3 (range 1–16.3)	30.4 days	Oral prednisolone 1 mg/kg/day for 5–7 days	96.2% (51/53, H–B grade II)	H–B grade
Karatoprak et al. (2019) [22]	Retrospective cohort	102 children with BP	10.37 (range 2–17)	6 months	Oral prednisolone 1–2 mg /kg/day for 5 days, gradually tapered for 10 days	99.0% (101/102, H–B grade I)	H–B grade
Arican et al. (2017) [3]	Retrospective cohort	88 children with BP	10.37 (range 0–18)	6 months	Oral prednisolone 1 or 2 mg/kg/day for 5 days, gradually tapered for 10 days	82.9% (73/88, H–B grade I)	H–B grade
Achour et al. (2015) [24]	Retrospective cohort	37 children with BP	13.9	Not specified	Oral prednisolone 1 mg/kg/day for 5–10 days	94.6% (35/37, H–B grade I)	H–B grade
Wang et al. (2010) [25]	Retrospective cohort	30 children with BP	8 (range 0–18)	12 months	Oral prednisolone 1 mg/kg/day or <1 mg/kg/day for 7 days	96.7% (29/30, H–B grade I)	H–B grade
Shih et al. (2009) [5]	Retrospective cohort	44 children with BP	6.9 (range 0–15)	3 months	Oral prednisolone 1 mg/kg/day for 7–10 days	97.7% (43/44)	Clinical evaluation
Chen and Wong (2005) [26]	Retrospective cohort	32 children with BP (3 recurrent attacks)	6.6 (range 0–15.5)	Not specified	Oral prednisolone 1–2 mg/kg/day for 14 days	96.9% (31/32)	Not specified
Tanaka et al. (2004) [27]	Retrospective cohort	38 children with BP	6.8	6 months	Oral prednisolone 0.5–1 mg/kg/day	100% (38/38)	Yanagihara scale or clinical evaluation

BP = Bell’s palsy; H–B grade = House–Brackmann grade.

## Data Availability

The data presented in this study are available on request from the corresponding author. The data are not publicly available as they were collected using clinical records.
